# Tectonic control on the persistence of glacially sculpted topography

**DOI:** 10.1038/ncomms9028

**Published:** 2015-08-14

**Authors:** Günther Prasicek, Isaac J. Larsen, David R. Montgomery

**Affiliations:** 1 Department of Geoinformatics, University of Salzburg, Hellbrunnerstraße 34, Salzburg 5020, Austria; 2 Division of Geological and Planetary Sciences, California Institute of Technology, MC-170-25, Pasadena, California 91125, USA; 3 Department of Earth and Space Sciences, University of Washington, Johnson Hall, 4000 15th Avenue NE, Seattle, Washington 98195, USA

## Abstract

One of the most fundamental insights for understanding how landscapes evolve is based on determining the extent to which topography was shaped by glaciers or by rivers. More than 10^4^ years after the last major glaciation the topography of mountain ranges worldwide remains dominated by characteristic glacial landforms such as U-shaped valleys, but an understanding of the persistence of such landforms is lacking. Here we use digital topographic data to analyse valley shapes at sites worldwide to demonstrate that the persistence of U-shaped valleys is controlled by the erosional response to tectonic forcing. Our findings indicate that glacial topography in Earth's most rapidly uplifting mountain ranges is rapidly replaced by fluvial topography and hence valley forms do not reflect the cumulative action of multiple glacial periods, implying that the classic physiographic signature of glaciated landscapes is best expressed in, and indeed limited by, the extent of relatively low-uplift terrain.

## Abstract (web summary)

U-shaped glacial valleys dominate >10 ka since the last major glaciation and the transitions from glacier-dominated to fluvial regimes are poorly understood. Here, the authors use digital topographic data to show that glacial topography is rapidly replaced by fluvial topography where rock uplift rates are high.

## Graphical abstract

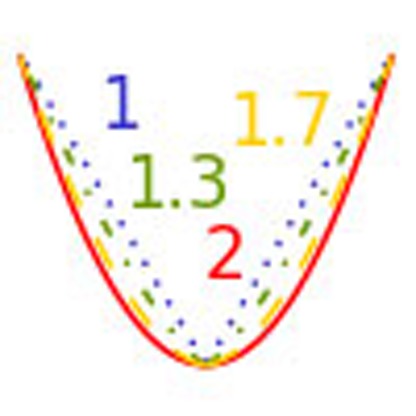

It has been recognized for over a century that alpine landscapes featuring horns, knife-edged ridges and U-shaped valleys are commonly associated with glacial sculpting^1^,^2^,^3^, whereas fluvial erosion is known to produce V-shaped valleys via links between river incision and landsliding^4^,^5^. Rivers, landslides and glaciers are all capable of rapid erosion at rates comparable to the highest rates of rock uplift^6^, and there has been progress in understanding how landscapes respond to the onset of glaciation^7^,^8^,^9^,^10^, how climate and tectonics influence erosion and topography in glacial landscapes^3^,^11^,^12^,^13^ and how landscapes react to deglaciation^14^,^15^. Less clear is the role of tectonic activity, and the pace at which fluvial incision and landsliding transform glacially carved topography into V-shaped valleys. Understanding this transition has implications for understanding the longevity of Earth's alpine landscapes, the degree to which glacial ‘preconditioning' can influence glacial extent and erosion in subsequent glaciations^7^ and our ability to assess the role glacial erosion has played in some of Earth's most rapidly uplifting mountains^16^.

Here we test whether the coupled fluvial and hillslope erosional response to tectonic forcing controls the timescale over which glacial topography persists into interglacial periods. We quantify the degree of glacial imprint on topography by analysing valley cross-sectional shapes across spatial gradients in rock uplift and erosion rates in mountain ranges worldwide to assess how tectonic forcing has influenced valley morphology and thus the transition from glacial to fluvial topography. For this we assume a flux steady state, such that rock uplift and erosion rates remain in balance with the accretionary flux^17^ throughout glacial–interglacial cycling. We also assume that glaciers carved U-shaped cross-sections throughout the study areas during the last glacial maximum (LGM). Valley cross-sections are automatically extracted from a digital terrain model and a power law is fitted to each side of the cross-section to quantify a ‘glaciality index' based on the shape of the valley flank, where an exponent of 1 indicates straight valley flanks forming a V-shaped valley cross-section and greater exponents are indicative of progressively more U-shaped valleys^18^,^19^(Fig. 1). Our analysis includes portions of landscapes covered with ice during the LGM but modern-day glaciers, lakes and large (> 5 km^2^) alluvial valley fills determined from published sources or manual mapping are excluded as they are likely to produce very high exponents due to a sharp transition from the valley bottom to the valley flanks.

We find that in Earth's most rapidly uplifting mountain ranges the lifespan of glacial topography is on the order of one interglacial period, which prevents the glacial signal from evolving over multiple glacial cycles. In contrast, glacial landscapes persist between glacial periods in relatively low-uplift terrain. At the onset of glaciation more erosion is required to (re-)establish U-shaped valleys on pre-existing fluvial topography than to maintain U-shaped forms on an existing glacial landscape^20^,^21^. Hence, the interplay of glacial erosion and tectonic forcing governs the morphologic impact of glaciations on active orogens, not only through influencing their height^22^ but also by altering the spatial and temporal patterns of erosion during subsequent glacial periods via a link between rock uplift and valley cross-sectional shape.

## Results

### Study areas

The Southern Alps of New Zealand are the surface expression of an on-going oblique collision of the Australian and Pacific plates and serve as a test site due to the unique tectonic and climatic setting—a wide range of rock uplift rates within which some locations experienced substantial glaciation, whereas others remained ice free. The Westland study area, located west of the range crest and parallel to the Alpine Fault (Fig. 2a,b), was covered repeatedly by ice throughout the Pleistocene^23^, has little variation in lithology^24^, rock mass strength^25^, glacial history^23^, peak elevations and relief^26^, but an exceptional gradient in tectonic forcing, with rock uplift rates that vary from 1 to 10 mm per year (27,28). The Marlborough study area, located in the northeastern part of the South Island (Fig. 2a), was not glaciated during the Pleistocene^23^ and shows classic V-shaped valley topography. The Fiordland study area is located about 400 km south of Westland (Fig. 2a), was repeatedly covered by glaciers^23^, has rock uplift rates 20 times lower than the centre of the Westland study area and features well-developed glacial cirques and U-shaped valleys^8^.

Westland represents the initial test site with spatially homogenous climatic and lithological characteristics. We used Marlborough and Fiordland to determine power-law exponent end-members for fluvially V-shaped and glacially U-shaped valleys. We subsequently assessed whether tectonics and erosion similarly control valley morphology in prominent mountain ranges worldwide with variable lithology, relief and climate, including a spatially extended study of the Southern Alps.

### Tectonic forcing, relief turnover time and valley shape

Mean power-law exponents for Marlborough and Fiordland are 1.28 and 1.54, respectively, and represent fluvial and glacial topographic end-members. Power-law exponents in Westland vary spatially as a function of rock uplift rate^27^, decreasing from values >1.5 where rock uplift rates are 2 mm per year to values <1.3 where rock uplift rates reach 6 mm per year and attain a nearly constant value of 1.3 where rock uplift rates exceed 6 mm per year (Fig. 3a). These findings demonstrate that the glacial imprint on topography diminishes in proportion to the rock uplift rate, with the landform transformed entirely from U-shaped to V-shaped since the LGM where rock uplift and erosion rates are high. In addition to rock uplift and erosional response, valley scale relief in Westland controls the duration of glacial topography, resulting in power-law exponents that exponentially increase with relief turnover time (relief divided by erosion rate; Supplementary Fig. 2). When plotted for the rock uplift rate bins from Fig. 3a, relief turnover times >250 kyr characterize areas that preserve well-developed glacial valleys with glaciality index values >1.4 (Fig. 3b). Hence, the best-developed glacial valley morphology appears restricted to landscapes with low rock uplift rates (≤2 mm per year) and high relief, leading to long-turnover times. For rock uplift rates >2 mm per year, the glacial valley morphology was significantly modified in postglacial time.

Full postglacial transformation from U-shaped to V-shaped valleys only occurred where rock uplift rates exceed 6 mm per year. Given the timing of the LGM in Westland of 17.3 kyr (ref. 29) and assuming that erosion rates balance rock uplift rates of 6–10 mm per year^30^,^31^, these results indicate that erosion of ∼100 to ∼175 m of rock, or one-seventh to one-quarter of the average relief (Supplementary Fig. 1b) needs to be eroded from these valleys to transform them from a U shape into a V shape. The timescale for this transformation is approximately one-seventh to one-quarter of the relief turnover time, corresponding to 8–113 kyr in Westland, for rock uplift rates of 10–2 mm per year, respectively. Given rock uplift rates of ∼0.6 mm per year in Fiordland, the timescale for landscape transformation there is roughly 200–350 kyr.

Our results show that the pace of rock uplift is a first-order control on the persistence of glacial valley cross-sectional morphology in the western Southern Alps. Tectonic forcing and erosional response significantly influence the postglacial modification of glacial valleys. The coupled action of fluvial incision and hillslope response in erasing the glacial imprint is achieved by intensive landslide erosion^30^,^32^. In Westland, the glacial imprint is fully removed from areas with the fastest rock uplift rates and the shortest turnover times in less than a single glacial–interglacial period, whereas in Fiordland U-shaped valleys persist from one glaciation to the next. Hence, the classic physiographic expression of glaciated mountain landscapes is limited to the extent of ‘low-uplift' terrain.

Valley shape data from the extended Southern Alps study area (Supplementary Fig. 3) and from two additional rapidly uplifting but previously glaciated landscapes (the eastern and western Himalayan syntaxes; Supplementary Figs 4 and 5) likewise reveal an inverse relation between power-law exponents and tectonic forcing (Fig. 3c). As erosion rates^16^ increase, valley shapes in formerly glaciated portions of the Himalayan syntaxes and the Southern Alps approach those of the Central Range of Taiwan, which can be considered a predominantly fluvial landscape, as only the highest peaks were glaciated during the LGM^33^ (Supplementary Fig. 5) and valley shape exponents indicative of fluvial topography are invariant across an exceptional gradient in erosion rates. Hence, our findings from the western Southern Alps are broadly applicable and demonstrate that glacial terrain can be modified to a fluvial landscape on timescales shorter than one interglacial period.

## Discussion

Rock type, tectonically influenced rock weakening, climate and the timing of glacial occupation likely influence the style of valley shape and channel–hillslope coupling in mountainous landscapes, but if eroding one-seventh to one-quarter of the relief is a general requirement for a complete transition from a glacial to a fluvial landscape, we estimate that the duration of some of Earth's most prominent alpine topography ranges from 10 to 500 kyr, depending on rock uplift rates, erosion rates and relief (Fig. 4). Within the current interglacial, previously glaciated terrain in Earth's most rapidly uplifting mountain ranges including the western Southern Alps and the eastern Himalayan syntaxis experienced strong fluvial overprinting with power-law exponents around 1.35. The western Himalayan syntaxis also exhibits fluvial overprinting (Fig. 3c), but the region-averaged exponent is higher due to asynchronous glaciation relative to both the Northern Hemisphere and the eastern Himalaya^34^, with a much later glacial advance at 9–5 kyr and hence a shorter duration of ice-free conditions. In these high-uplift regions, a morphologic transition from glacial to fluvial terrain within one interglacial period prevents the development of an accumulated topographic signal over multiple glacial cycles. In contrast, in most alpine landscapes more than 100 kyr are required for the transformation, and glacial morphology has not or has only partially been erased during the current interglacial, as indicated by exponents of 1.4–1.5. The varying exponent values in these low-uplift mountain ranges are probably due, at least in part, to lithological and climatic influences. In addition, inner gorges may lower exponents of heavily glaciated terrain, as, for example, in the European Alps^35^. Variations in power-law exponents may also emerge from uncertainties in mapping of modern glaciers, alluvium, lakes and LGM ice extent.

Numerical simulations indicate that the presence of glacially modified terrain when ice builds up during a cooling climate drastically influences glacial extent and erosion^7^. In particular, pre-existing glacial terrain facilitates highly nonlinear and rapid glacial expansion when the equilibrium line altitude falls below the hypsometric maximum. In contrast, in fluvial landscapes experiencing first-time glaciations, glacial area and climate are nearly linearly related, leading to an earlier onset of glacial occupation and higher overall glacial erosion^7^. More intensive topographic modification is required to (re-)establish a U-shaped valley cross-section on fluvial terrain than to maintain it on pre-existing glacial topography. In low-uplift mountain landscapes, this is indicated by exceptional periods of rapid erosion coincident with the onset of pronounced Pleistocene glaciation. The Coast Mountains of British Columbia experienced a sixfold increase in erosion rates up to 5 mm per year during the onset of extensive glaciation about 1.8 Myr ago, whereas total erosion has been ≤300 m since 1.4 Myr (ref. 21), as ice has presumably been re-occupying persistent U-shaped terrain. Fiordland responded similarly, with rapid landscape modification at the onset of glaciation but little change in the last 1.5 Myr (ref. 8). In addition, valley shape also influences the mechanism of erosion. Bedrock stresses beneath glacial ice in an initial V-shaped topography induce extensional fracturing and drive rapid U-shaped valley development via enhanced glacial quarrying. Without extensive erosion during interglacial periods, differential stress and extensional fracturing decrease in subsequent glacial cycles leading to a reduction in topographic modification^20^. We therefore suggest that slowly uplifting mountain ranges experienced a strong topographic control on ice extent and glacial erosion during the most recent Quaternary glaciations expressed in a cumulative (or polycyclic) glacial signal, whereas a lack of pre-existing glacial topography during the onset of glacial periods increased glacial erosion in Earth's most rapidly uplifting mountain ranges. Thus, our results imply that tectonic forcing governs the impact of glaciations on active orogens beyond controlling their height via enhanced ice coverage at high elevations^22^. The effect of the pace of tectonic forcing on valley erosion provides a link between rock uplift and valley cross-sectional shape that influences landscape-scale erosion rates by altering the topography that glaciers encounter and erode as climate cools.

Tectonic forcing is a first-order control on landscape evolution and on the persistence of glacial morphology. In the Earth's most rapidly uplifting mountain ranges the lifespan of glacial topography is on the order of one interglacial period. The short lifespan prevents the development of a topographic signal that accumulates over multiple glacial cycles and instead causes valleys to oscillate between a U- and V-shape during a succession of glacial and interglacial periods. This modulates the spatial and temporal distribution of glacial occupation and increases erosion rates when ice returns to the landscape, relative to landscapes where glacial topography persists between glaciations. The influence of tectonic forcing on the transition from a glacial to a fluvial mountain landscape and the implications for erosion during glacial and interglacial periods play important roles in determining the nature of the links among climate, erosion and topography in mountains. In short, the world's classic glacially sculpted terrain formed over multiple glaciations in orogens with low-uplift rates, whereas formerly glaciated, but rapidly uplifting mountains likely experienced enhanced glacial erosion throughout the Quaternary, despite now lacking the topographic signature of glaciation.

## Methods

### Valley extraction

Valley cross-sections were extracted automatically from digital elevation models (DEMs) to facilitate this large-scale analysis. The New Zealand National Digital Elevation Model has a resolution of ∼25 m and was used for the Westland, Fiordland and Marlborough study areas. For the global study, we used the Aster GDEM 2 with a cell size of 1″,which was projected to a resolution of 25 m to allow comparability of the results. We excluded modern-day glaciers, lakes and large (> 5 km^2^) alluvial valley fills from the New Zealand study areas, using lake and glacier data from Land Information New Zealand and manual mapping of alluvium. For all other study areas, alluvium and lakes were manually mapped and modern-day glaciers were extracted from the GLIMS Glacier Database^36^.

For each DEM, valley-bottom flow paths (thalweg) were defined by calculating flow accumulation using a 1-km^2^ drainage area threshold. One valley cross-section was extracted for each flow path grid cell, resulting in a large number of cross-section analyses for each valley—a total of 2.5 million cross-sections were analysed with upstream drainage areas ranging from 1 to ∼2,600 km^2^. For each flow path grid cell, we defined the orientation of the valley cross-section and the grid cells composing it by using an algorithm that automatically adjusts the length of the analysis scale to match the valley cross-sectional width^19^. This adjustment is based on multi-scale curvature and drainage area constraints; minimum curvature is extracted over multiple moving window sizes ranging from the close vicinity of the flow path to those exceeding the largest valleys in the study areas. For U-shaped valleys, curvature is the most concave when calculated for a length scale that spans the entire valley cross-section. For V-shaped valleys, concavity is relatively scale invariant. However, for both cases, curvature declines once the length of the analysis scale is large enough to overlap with ridges or prominent valley shoulders (Supplementary Fig. 7). Hence, the variable analysis length scale with the most concave curvature was consequently selected to represent the valley scale^19^ and this valley cross-section was extracted for calculation of the glaciality index.

### Glaciality index

**Figure 1:**
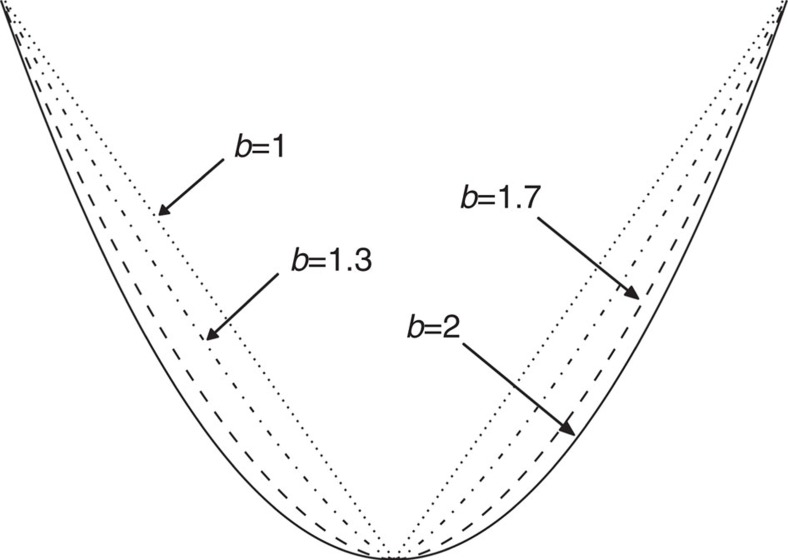
Fitting power laws to valley cross-sections. Power laws of the form *y*=*ax*^*b*^ fit to valley flanks with the exponent *b* depicting their shape. *b*=1 represents the straight valley flanks of a V-shaped valley and greater exponents are indicative of progressively more U-shaped valleys.

Each extracted cross-section was automatically split at its lowest elevation point and this location was then set to be the origin (*x*=0, *y*=0) of the local coordinate systems of each side of the cross-section^37^. A power law of the form *y*=*ax*^*b*^ was fit to each side of the cross-section (each valley flank) by nonlinear least-squares using Scientific Python^38^,^39^. The mean of the exponents of the two power laws was used to quantify a ‘glaciality index' keyed to the shape of the valley flank, where an exponent of 1 indicates straight valley flanks that form a V-shaped valley cross-section and progressively greater exponents are indicative of progressively more U-shaped valleys^18^ (Fig. 1). Mean *R*^2^ values are 0.98, 0.97 and 0.97 for power laws fitted to valley cross-sections extracted from the Marlborough, Fiordland and Westland study areas, respectively. In all three study areas, *R*^2^ values are >0.99 for ∼30% of the power-law fits.

### Relief turnover

Relief turnover time (Fig. 3b) is calculated as valley relief divided by erosion rate. To calculate valley relief, we first isolate DEM grid cells on ridge lines. We then interpolate between those points using an inverse distance weighted interpolation^40^. Valley relief is calculated as the vertical distance between the interpolated ridge surface and the flow path grid cells.

**Figure 2:**
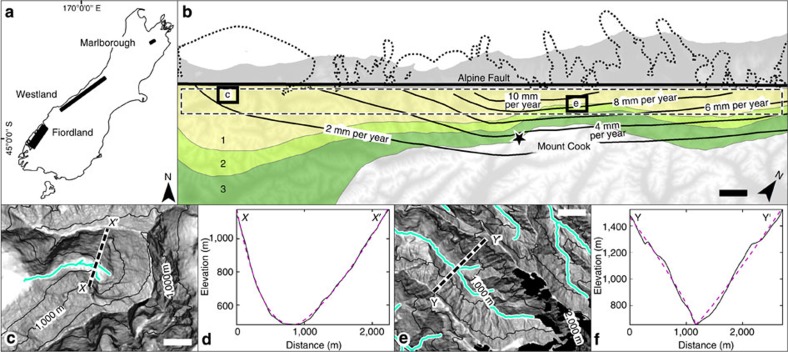
Study area characteristics. (**a**) Location of the main study area in Westland and the two reference study areas, Marlborough (fluvial) and Fiordland (glacial) on New Zealand's South Island. (**b**) Extent of Westland study area (dashed rectangular outline), LGM extent^23^ (dotted outline), modern coastline (grey shading), rock uplift rates^27^ (solid contour lines), geological map showing metamorphic grade of Alpine schist south of the Alpine Fault, Garnet-Oligoclase zone (1), Biotite zone (2), and Chlorite zone (3) (ref. 24), plotted on a digital terrain model. Bold black rectangles outline the extents of subfigures **c** and **e**. Scale bar, 10-km wide. (**c**) Shaded relief map and an example cross-section (dashed line; *X*–*X*′) for strongly glacial, low-uplift terrain in the Westland study area. Scale bar, 1-km wide. (**d**) Example cross-section (*X*–*X*′; solid line) and fitted power law (dashed line, *b*=1.95). (**e**) Shaded relief map and example cross-section (dashed line; *Y*–*Y*′) of strongly fluvial, high-uplift terrain in the Westland study area. Black polygons delimit current ice cover and are not included in analysis. Scale bar, 1-km wide. (**f**) Example cross-section (*Y*–*Y*'; solid line) and fitted power law (dashed line, *b*=0.97). Contour spacing in **c** and **e** is 500 m, cyan lines show flow path cells for valley cross-section extraction.

**Figure 3:**
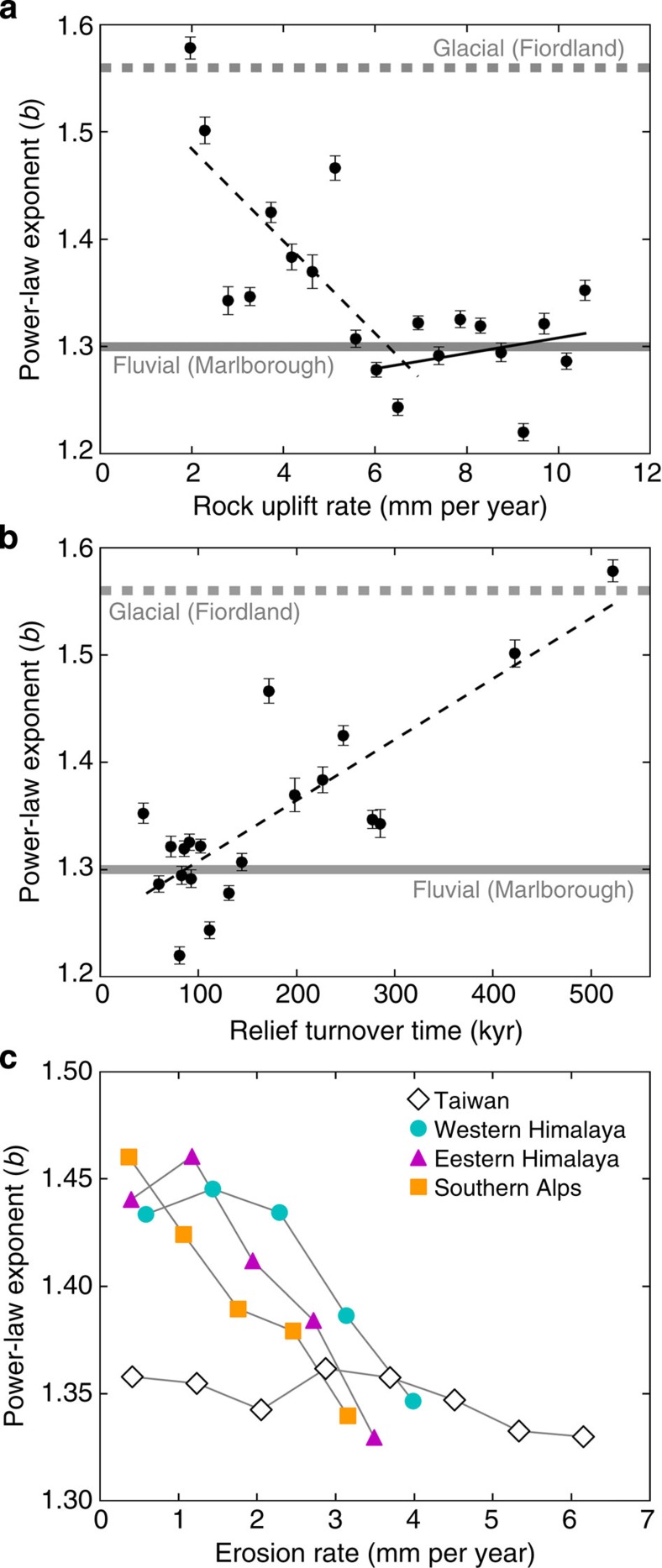
Cross-sectional valley shape, turnover time, rock uplift and erosion rates. (**a**) Mean power-law exponents for 20 rock uplift^27^ bins from Westland (bin size=0.45 mm per year). Regression lines fitted separately to data with a rock uplift rate <6 mm per year (dashed line, *y*=−0.043 (±0.002)*x*+1.569(±0.011), *R*^*2*^=0.54, root-mean-squared error (r.m.s.e.)=0.06, *P*<0.01) and >6 mm per year (solid line, *y*=0.007 (±0.002)*x*+1.236(±0.014), *R*^*2*^=0.08, r.m.s.e.=0.04, *P*=0.4). Reference exponents: fluvial from Marlborough (1.28; solid grey line) and glacial from Fiordland (1.54; dashed grey line). Error bars indicate±1 s.e. See Supplementary Discussion and Supplementary Fig. 1 for further information on data distribution and statistics. (**b**) Mean power-law exponents of 20 rock uplift^27^ bins from Westland plotted against relief turnover time. Turnover time is defined as relief/erosion rate and we assume rock uplift rates equal erosion rates. Power-law exponent increases with turnover time as *y*=0.001(±<0.001)*x*+1.251(±<0.001), *R*^*2*^=0.67, r.m.s.e.=0.05, *P*<0.01. (**c**) Power-law exponents plotted against erosion rates for Earth's most rapidly uplifting and eroding but previously glaciated landscapes and one landscape with little glacial modification (Taiwan). A gridded erosion rate data set derived from thermochronology^16^ that is common to all four regions is used as a rock uplift rate proxy. For study area maps, see Supplementary Figs 3–5.

We assume a flux steady state^17^, such that the erosion rate equals the rock uplift rate, as the the Southern Alps have long been recognized as a landscape where rock uplift and erosion are balanced^31^. Rock uplift rates for the western Southern Alps (Fig. 3a,b) are based on published rock uplift contours derived from thermochronology data^27^. The rock uplift contours (Fig. 2b) were interpolated to a raster data set without drainage enforcement.

### Rock uplift and erosion rate data

Long-term erosion rate data based on a global inversion of thermochronology data^16^ were used to assess the relationship between valley shape and erosion rate for New Zealand, the Himalayan syntaxes and Taiwan (Fig. 3c). The gridded point erosion rates^16^ were interpolated to a 25-m raster using a bilinear interpolation technique. The area used in the analysis was restricted to either the extent of the gridded erosion rate data or the extent of well-developed relief (Supplementary Figs 3–5). The rock uplift^27^ (Fig. 3a,b) and erosion rate^16^ (Fig. 3c) data from the western Southern Alps exhibit similar spatial gradients, but differ in absolute magnitude, as discussed further in the Supplementary Discussion (Comparison of rock uplift and erosion rate datasets) and in Supplementary Fig. 8.

**Figure 4:**
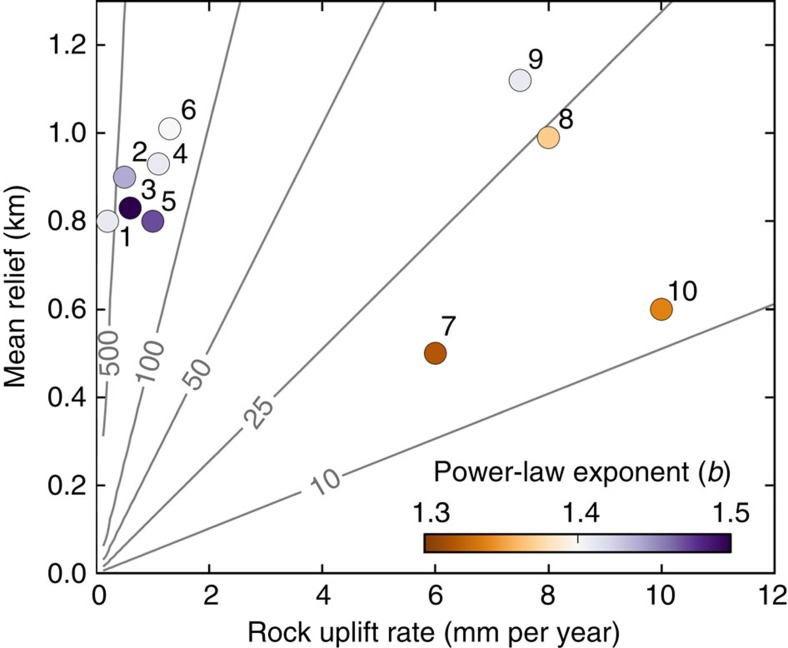
A global perspective on tectonics and valley shape. (**a**) Rock uplift plotted against average relief of some of Earth's major mountain ranges: Washington Cascades^41^, WA, USA (1); Coast Mountains^42^, BC, Canada (2); Fiordland^8^, New Zealand (3); Greater Caucasus^43^, Georgia/Russia (4); Northern Patagonian Andes^44^, Chile (5); European Alps^45^, Austria/Italy/Switzerland (6); Central Range^46^, Taiwan (7); Eastern Himalaya^47^, China (8); Western Himalaya^48^, Pakistan (9); and High-uplift Westland^27^, New Zealand (10). Contours indicate time (kyr) necessary to renew 20% of the relief, the average fraction of relief turnover time required to transform a U-shaped into a V-shaped valley in Westland. The minimum, mean and maximum durations of the last four interglacial periods are 20, 22 and 26 kyr, respectively^49^, indicating that transformation from glacial to fluvial landscape morphology can occur during a single interglacial period for comparable or lower contour values. Power-law exponents indicate the degree of glacial imprint on topography. See Supplementary Fig. 6 for study area maps.

Alpine landscapes of some of earth's major mountain ranges (Fig. 4) were analysed using published exhumation rate data^8^,^27^,^41^,^42^,^43^,^44^,^45^,^46^,^47^,^48^, which we assume to reflect rock uplift. The size of the areas used in the analyses are limited by the extent of the highest rock uplift rates (Himalaya, New Zealand), the extent of past and recent glaciation, the extent of well-developed relief and/or computational feasibility (Supplementary Fig. 6).

## Additional information

**How to cite this article:** Prasicek, G. *et al.* Tectonic control on the persistence of glacially sculpted topography. *Nat. Commun.* 6:8028 doi: 10.1038/ncomms9028 (2015).

## Supplementary Material

Supplementary Information Supplementary Figures 1-8, Supplementary Discussion and Supplementary References
