# Supplementary material for: Tectonic control on the persistence of glacially sculpted topography
Source: Nat Commun. 2015 Aug 14;6:8028. doi: 10.1038/ncomms9028 (PMC4557346; doi:10.1038/ncomms9028)
Supplement: Supplementary Information — Supplementary Figures 1-8, Supplementary Discussion and Supplementary References [file ncomms9028-s1.pdf]

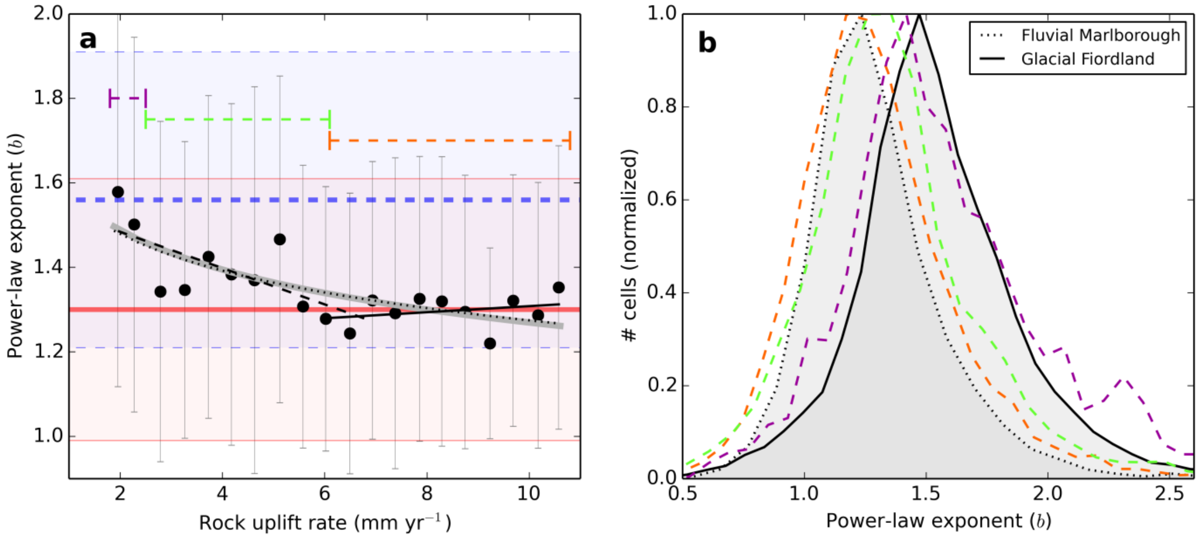

Supplementary Figure 1 | Power-law exponent distribution in Westland. a, Mean power-law exponents for 20 rock uplift bins from Westland. Error bars indicate ± 1 SD of the power-law exponents per rock uplift bin. Linear regression lines (black) fitted separately to bins with a rock uplift rate < 6 mm yr^{-1} (dashed line) and > 6 mm yr^{-1} (solid line). Power-law regression lines fitted to the bin means (black, dotted, $y = 1.59(\pm 0.053)*x^{-0.1\ (\pm 0.02)}$, $R^2 = 0.6$, $RMSE = 0.06$, $P = 0.05$) and to the entire distribution (bold gray, $y = 1.60(\pm 0.008)*x^{-0.1(\pm 0.003)}$, $R^2 = 0.03$, $RMSE = 0.36$, $P < 0.01$) indicate that the bin means represent the entire distribution well. Reference exponents: fluvial from Marlborough (1.28; bold solid red line) ± 1 SD (0.94 and 1.62; thin solid red lines and red shading) and glacial from Fiordland (1.54; bold dashed blue line) ± 1 SD (1.19 and 1.89; thin dashed blue lines and blue shading). **b**, Histograms of three color-coded rock uplift rate bins shown in (a).

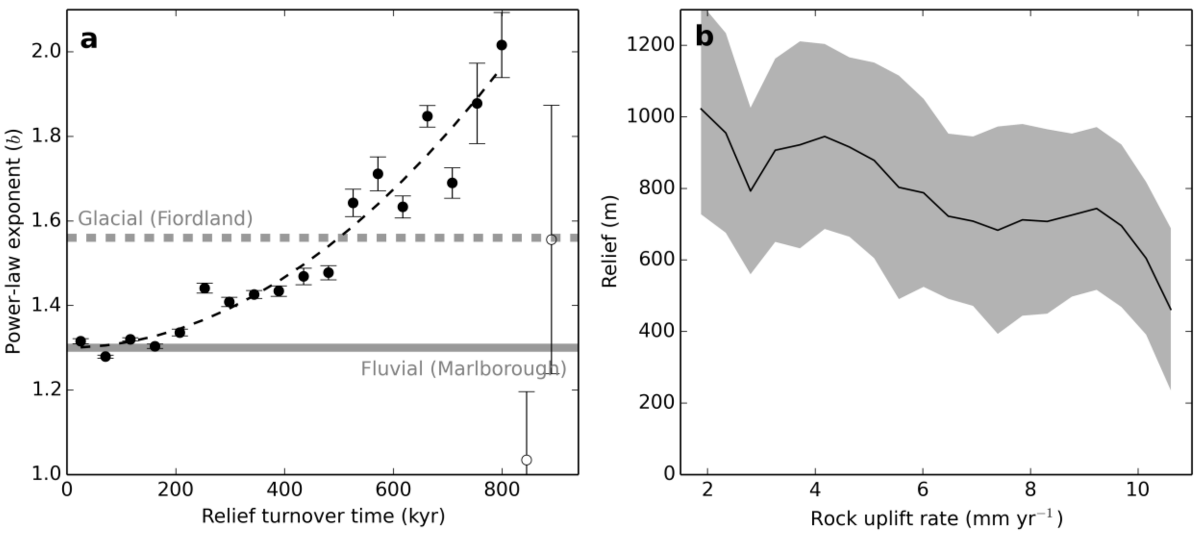

Supplementary Figure 2 | Relief and relief turnover time in Westland. a, Mean power-law exponents plotted for 20 relief turnover time bins. Exponents decrease with increasing relief and decreasing rock uplift rates. Power-law regression line fitted to mean exponents of bins with sample sizes from 60 to 8000 (black circles, $y = 0.0001\ (\pm 0.0001) * x^{1.99\ (\pm 0.22)}$, $R^2 = 0.94$, $RMSE = 0.05$, $P < 0.01$). White circles with $n < 10$ are omitted from the power-law fit. Error bars indicate ± 1 SE. **b**, Relief plotted against rock uplift rate for flow path cells of the Westland study area. Gray area depicts ± 1 SD. Relief decreases with rock uplift and is greatest for low rock uplift rates where glacial landforms prevail.

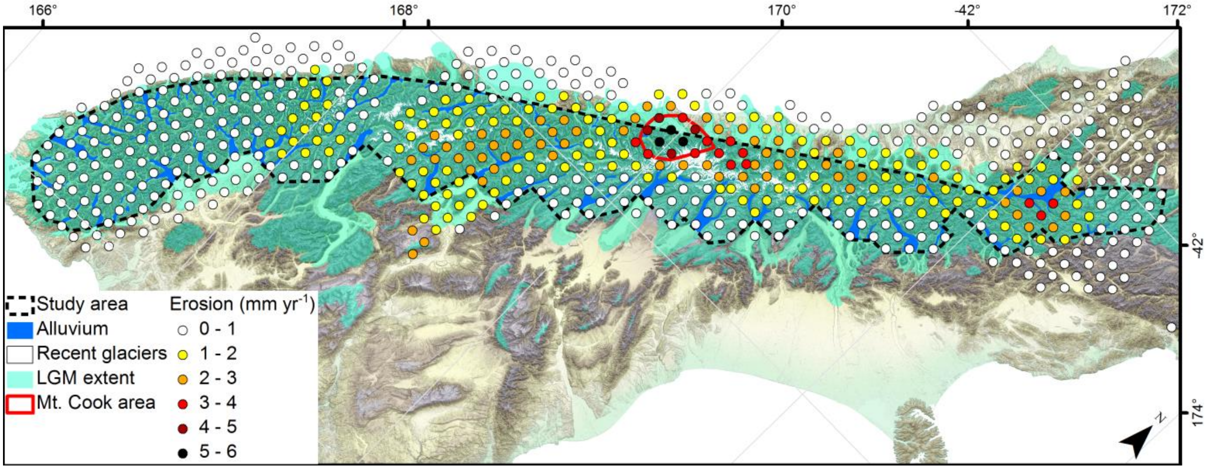

Supplementary Figure 3 | Study area from the New Zealand Southern Alps. The study area was used to generate data in Fig. 3c. It is restricted by the extent of the gridded erosion rate data[1]or well-developed relief. Only exponents derived from cells within the LGM limits[2] were used. The area outlined in red was excluded from the data in Fig. 3c, but is shown in Supplementary Fig. 8d. See supplementary text for discussion.

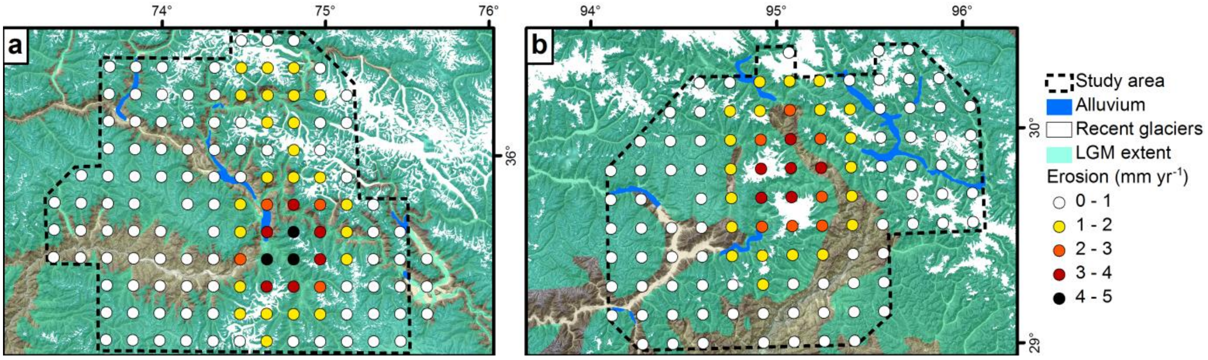

Supplementary Figure 4 | Himalayan study areas. Study areas from Western (a) and Eastern (b) Himalaya used to generate the data in Fig. 3c. The study sites are restricted by the extent of the gridded erosion rate data[1]. In both cases only exponents derived from cells within the LGM limits were used[3,4].

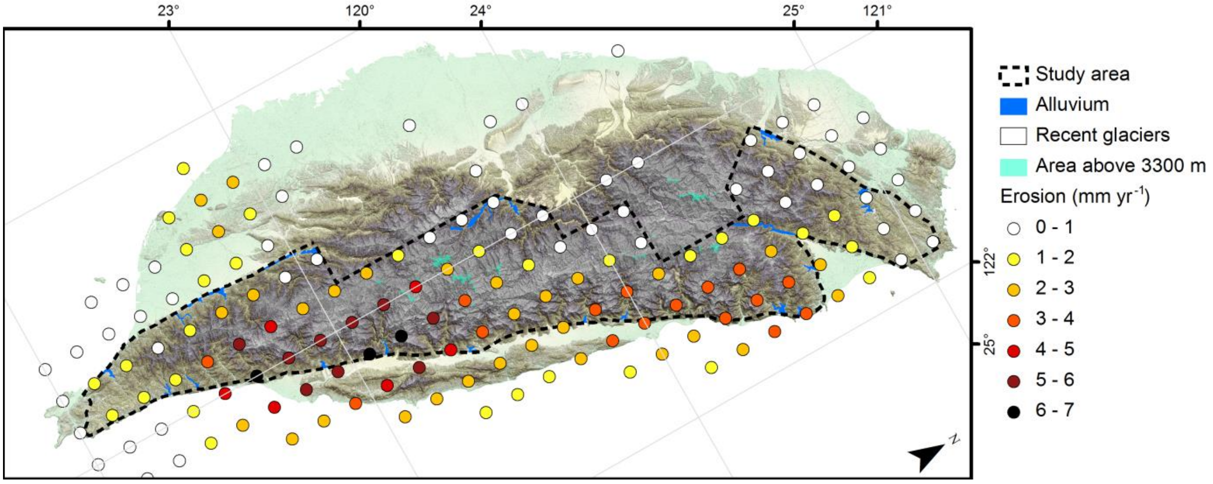

Supplementary Figure 5 | Study area from Taiwan. The study area was used to generate data in Fig. 3c. It is restricted by the extent of the gridded erosion rate data[1] and by the area with well-developed relief. LGM glacier extent is not well constrained but the LGM ELA has been reconstructed[5] and is located at approximately 3300 m. This leaves only the highest peaks of Taiwan under glacial influence during the LGM. Areas above and below this altitude have nearly identical power-law exponents (1.35 and 1.33, respectively), indicative of fluvial topography. Therefore, the extent of the gridded erosion rate data was used to extract exponents for Taiwan in Figs. 3c and 4.

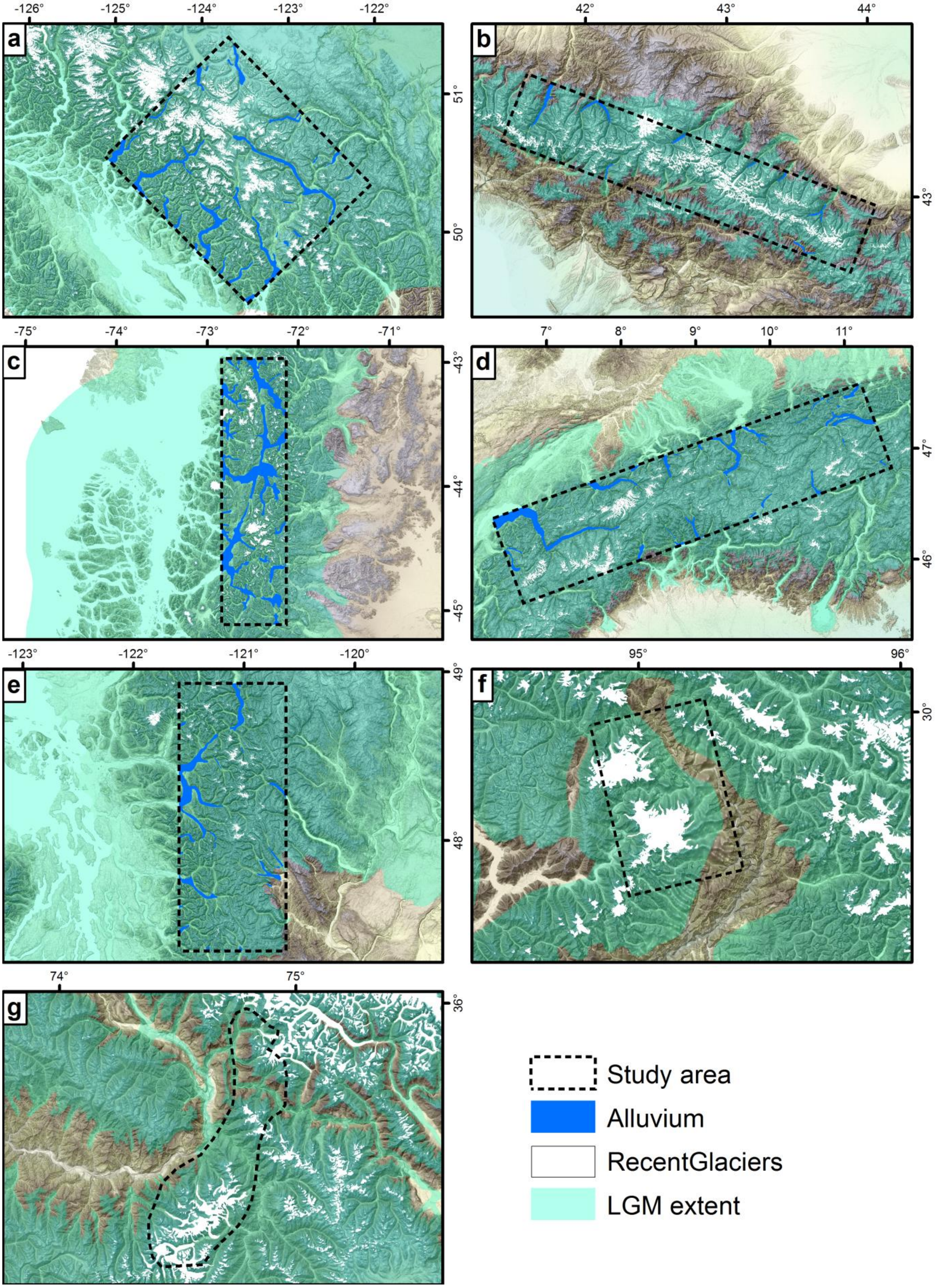

Supplementary Figure 6 | Study areas used to generate data for Fig. 4. Only exponents derived from cells within the LGM limits were used and recent glaciers and large depositional

forms like alluvium were excluded. a) Coast Mountains, BC, Canada; b) Greater Caucasus, Georgia/Russia; c) Northern Patagonian Andes, Chile; d) Central Alps, Austria, Italy, Switzerland; e) Cascades, Washington, US; f) Eastern Himalaya, China; g) Western Himalaya, Pakistan;. Sample areas are either confined by the limited extent of highest rock uplift rates combined with the extent of past and recent glaciation (Eastern Himalaya[3,6], Western Himalaya[4,7], New Zealand[2,8]), or by the extent of past and recent glaciation and computational feasibility (Coast Mountains[9], Greater Caucasus[10], Patagonian Andes[11], Central Alps[12,13,14], Cascades[15]).

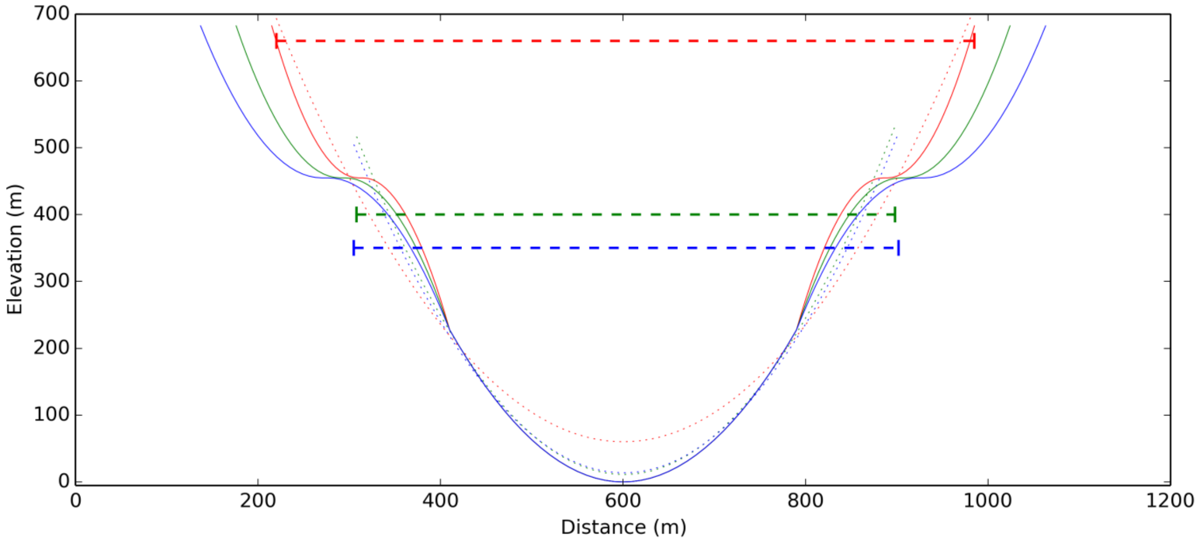

Supplementary Figure 7 | Exclusion of valley shoulders. Idealized glacial valley cross sections with valley shoulders of varying prominence (solid lines). Dotted lines represent fitted second-order polynomials yielding the most concave curvature values for each cross section which define the extent of the analysis scales (dashed lines). Prominent shoulders (green, blue) lead to an exclusion of shoulders and superior terrain.

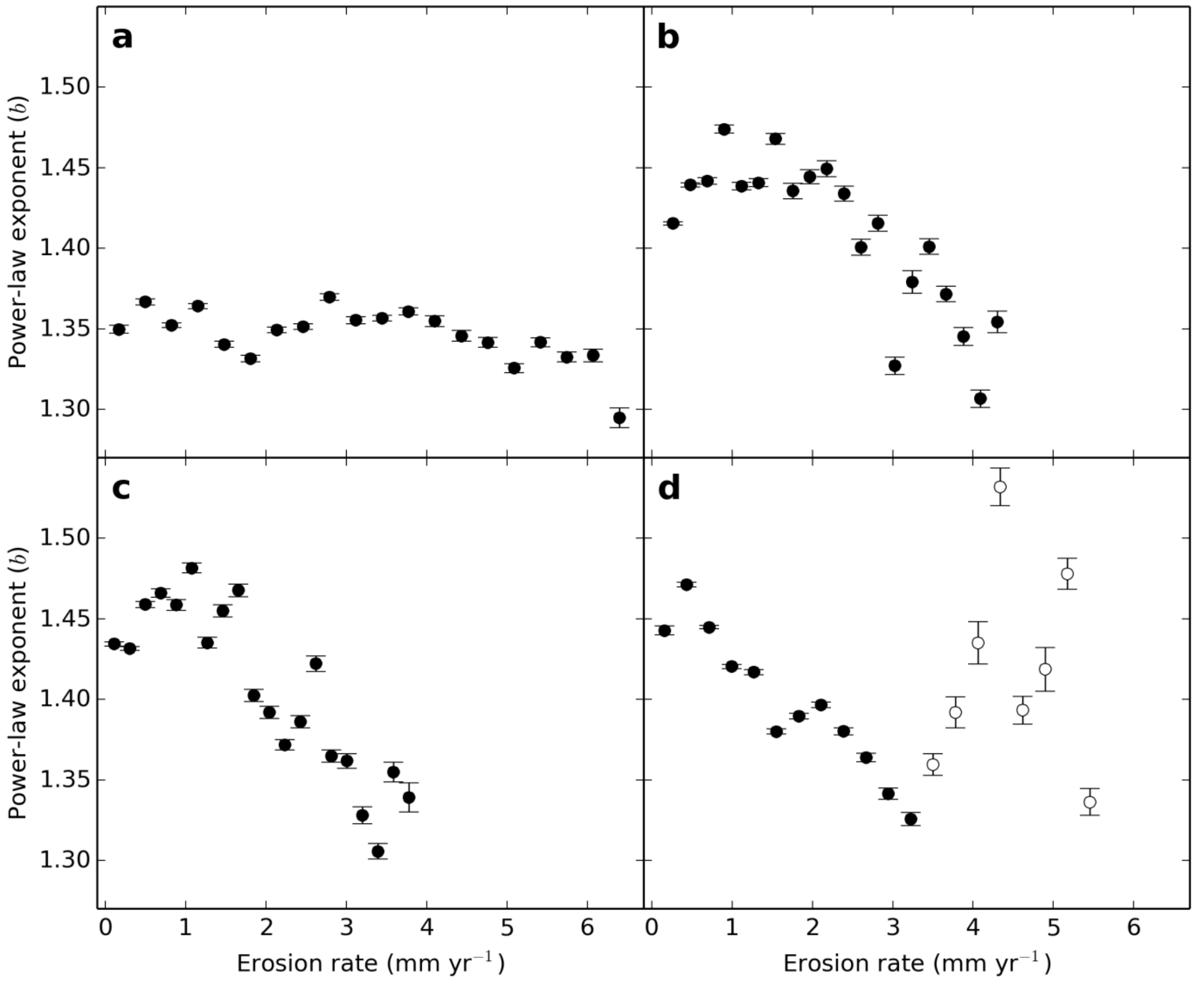

Supplementary Figure 8 | Power-law exponents for rapidly uplifting mountain ranges. Power-law exponents plotted against 20 erosion rate[1] bins for Taiwan (a), western Himalaya (b), eastern Himalaya (c), and New Zealand (d) including the immediate vicinity of Mt. Cook (white circles; red polygon in Supplementary Fig. 3). Error bars indicate ± 1 SE. See text for discussion.

Supplementary Discussion

Variability in cross-sectional valley shape

Approximately 2.5 million valley cross sections were automatically extracted from digital elevation models for this study. Mean exponents of power-laws fitted to these cross-sections plotted against bins of rock uplift or erosion rates define robust trends. The mean power-law exponents derived from our automated, global analysis are slightly lower than, but generally in good agreement with those found in the literature[16,17,18]. For example, a mean exponent of 1.87 (min = 1.03, max = 3.5) was found for 49 valley cross profiles in the Tien Shan Mountains[19]. While we identified large glacial valleys with long turnover time to have the highest exponents (Supplementary Fig. 2), our study sites are dominated by smaller valleys, which explains the lower mean exponents found with our approach. However, our power-law exponents have high variability in both glacial and fluvial landscapes (Supplementary Fig. 1). Standard deviations are high for the 20 rock uplift bins in Supplementary Fig. 1a, but the histograms in Supplementary Fig. 1b show nonetheless that the entire distributions of power-law exponents shift towards higher exponents with decreasing rock uplift rates. To further test whether the large scatter is due to natural variability of cross-sectional valley shape or due to methodological issues we compared the distribution of the 3000 exponents automatically derived from fluvial Marlborough with 52 manually extracted cross sections from the same set of thalweg cells. For this, we randomly selected flow path cells to serve as the center of the manually defined cross sections. Both mean (*m*) and standard deviation (*std*) of the manual test data (*m* = 1.31., *std* = 0.40) deviate only slightly from the automatically derived exponents (*m* = 1.28, *std* = 0.34). This shows that the large scatter arises from natural variability in cross-sectional valley shape, regardless of whether cross sections are drawn for all flow path cells (automated) or for randomly selected cells along the flow path (manual). Minimum and maximum exponents for Marlborough are 0.01 and 5.83, respectively. While

extreme exponents < 0.5 or > 2.5 constitute only 1.4 % of the exponent distribution in Westland, extracting one valley cross section for each flow-line cell is basically equivalent to analyzing the entirety of valley flank morphology present in a DEM and hence must result in considerable scatter. In particular, the influences of tributary confluences, roche moutonnées and rugged terrain on power-law exponents are difficult to constrain. However, mean exponents calculated for rock uplift or erosion bins show little variation in fluvial regimes even for large gradients in rock uplift or erosion. This is shown in Fig. 3c and Supplementary Fig. 8a, where mean exponents for Taiwan are close to 1.3 and have a very small standard error despite an exceptional gradient in erosion rates from 0.5 to 6 mm yr^{-1} (Supplementary Fig. 5). Hence, the large variability in cross-sectional valley shape does not compromise the ability of mean exponents to differentiate U-shaped and V-shaped topography.

DEM quality

A DEM resolution sufficient to resolve valley morphology is the most important technical prerequisite for our approach. For the initial study of glaciated terrain in Westland, a resolution of 25 m was chosen to provide sufficient data points for power-law fitting while keeping the analyses computationally feasible. For our analyses of mountain ranges worldwide, global data availability was an additional limitation. At the time when data processing was done, the Aster GDEM 2 was the only global DEM with a resolution similar to the New Zealand Digital Elevation Model and was therefore selected to allow comparability of the results. While resolutions are similar, differences in the accuracy of the two datasets[20,21] likely have a minor effect on the extracted power-law exponents. For example, mean exponents for Fiordland are 1.54 and 1.52 for the New Zealand Digital Elevation Model and the Aster GDEM 2, respectively.

Comparison of rock uplift and erosion rate datasets

The erosion rates[1] for Westland shown in Fig. 3c are much lower than rock uplift rates[7] reported for the same area (shown in Fig. 3a), which are generally in good agreement with other sources for Westland[22,23,24,25]. The erosion rates[1] are also generally lower in comparison to values reported in studies[6,7,8,26,27,28,29] for the regions shown in Figs. 3c (study areas depicted in Supplementary Figs. 3 to 5) and 4 (study areas depicted in Supplementary Fig. 6). The difference in magnitude may be due to the timescales involved or the techniques employed to create the gridded erosion rate dataset[1]. Despite the differences in magnitude, the spatial patterns of erosion[1] and rock uplift[8] are generally similar. Hence we use the erosion dataset as an indicator of the general long-term distribution of erosion rates that facilitates comparisons of spatial patterns among different study sites (e.g., Fig. 3c).

The exponent-rock uplift relation for Westland (Fig. 3a) differs from the exponent-erosion trend (Fig. 3c) due to differences in the rock uplift[8] and erosion[1] data sources. Valley shape exponents decrease with increasing erosion rates from 0.3 to about 3.5 mm yr^{-1}, a relationship that holds for 98% of the study area (Supplementary Fig. 3). However, for the highest erosion rates in the erosion dataset the exponents increase in the vicinity of Mt. Cook (Supplementary Fig. 8), in an area that constitutes about 2% of the study area but hosts an erosion rate gradient from 3.5 to about 5.5 mm yr^{-1}. The Mt. Cook region experienced stronger post-LGM glacial advances than other parts of Westland[30], probably due to its exceptionally high topography. This may have prevented a full transition from U-shaped to V-shaped valleys since the LGM in this area. In addition, the limited spatial distribution of these high erosion rates in New Zealand differs from other studies where high rates extend over much more extensive areas[8,22]. The zone with high erosion rates near Mt. Cook arises from only a few erosion grid cells, some which are located northwest of the Alpine Fault (the downthrown block) that are anomalously portrayed as having erosion rates > 3.5 mm yr^{-1}. A gridding artifact may be

responsible for the anomalous positioning of some of the high erosion rate grid cells, as this area accounts for little more than a single 3 x 3 cell neighborhood quadrat on the erosion rate grid, the standard neighborhood used for raster data manipulation. We suspect that the anomalous exponent-erosion rate trend for the Mt. Cook area shown in Supplementary Fig. 8 results either from differences in glacial history or a gridding effect. Thus, we have omitted this region from the analysis used to generate Fig. 3c.

Supplementary References

1 Herman, F. *et al.* Worldwide acceleration of mountain erosion under a cooling climate. *Nature* **504**, 423-426 (2013).

2 Barrell, D. J. A. in *Developments in Quaternary Sciences* Vol. 15 (eds J. Ehlers, P. L. Gibbard & P. D. Hughes) 1047-1064 (Elsevier, 2011).

3 Li, B., Li, J., Cui, Z. *Quaternary glacial distribution map of Qinghai-Xizang (Tibet) Plateau*. (Lanzhou University, 1991).

4 Kamp, U. & Haserodt, K. in *Developments in Quaternary Sciences* Vol. 2, Part C (eds J. Ehlers & P. L. Gibbard) 293-311 (Elsevier, 2004).

5 Böse, M. in *Developments in Quaternary Sciences* Vol. 2, Part C (eds J. Ehlers & P. L. Gibbard) 347-352 (Elsevier, 2004).

6 Larsen, I. J. & Montgomery, D. R. Landslide erosion coupled to tectonics and river incision. *Nature Geoscience* **5**, 468-473 (2012).

7 Koons, P. O., Zeitler, P. K. & Hallet, B. in *Treatise on Geomorphology* (ed John F. Shroder) 318-349 (Academic Press, 2013).

8 Tippett, J. M. & Kamp, P. J. J. Fission track analysis of the Late Cenozoic vertical kinematics of continental pacific crust, South Island, New Zealand. *Journal of Geophysical Research: Solid Earth* **98**, 16119-16148 (1993).

9 Fulton, R. J., Ryder, J. M. & Tsang, S. in *Developments in Quaternary Sciences* Vol. 2, Part B (eds J. Ehlers & P. L. Gibbard) 39-50 (Elsevier, 2004).

10 Gobejishvili, R. in *Developments in Quaternary Sciences* Vol. 2, Part A (eds J. Ehlers & P. L. Gibbard) 129-134 (Elsevier, 2004).

11 Coronato, A., Meglioli, A. & Rabassa, J. in *Developments in Quaternary Sciences* Vol. 2, Part C (eds J. Ehlers & P. L. Gibbard) 45-48 (Elsevier, 2004).

12 van Husen, D. in *Developments in Quaternary Sciences* Vol. 2, Part A (eds J. Ehlers & P. L. Gibbard) 1-13 (Elsevier, 2004).

13 Carraro, F. & Giardino, M. in *Developments in Quaternary Sciences* Vol. 2, Part A (eds J. Ehlers & P. L. Gibbard) 201-208 (Elsevier, 2004).

14 Schlüchter, C. in *Developments in Quaternary Sciences* Vol. 2, Part A (eds J. Ehlers & P. L. Gibbard) 413-418 (Elsevier, 2004).

15 Porter, S. C. in *Developments in Quaternary Sciences* Vol. 2, Part B (eds J. Ehlers & P. L. Gibbard) 289-293 (Elsevier, 2004).

16 Doornkamp, J. C. & King, C. A. M. *Numerical Analysis in Geomorphology*. (Arnold, 1971).

17 Graf, W. L. The Geomorphology of the Glacial Valley Cross Section. *Arctic and Alpine Research* **2**, 303-312 (1970).

18 Wheeler, D. A. Using parabolas to describe the cross-sections of glaciated valleys. *Earth Surface Processes and Landforms* **9**, 391-394 (1984).

19 Li, Y., Liu, G. & Cui, Z. Glacial valley cross-profile morphology, Tian Shan Mountains, China. *Geomorphology* **38**, 153-166 (2001).

20 Barringer, J. R. F., Pairman, D. & McNeill, S. J. *Development of a high-resolution digital elevation model for New Zealand*. (Landcare Research, 2002).

21 Gesch, D., Oimoen, M., Zhang, Z., Meyer, D., Danielson, J. Validation of the Aster Global Digital Elevation Model Version 2 over the conterminous United States.

International Archives of the Photogrammetry, Remote Sensing and Spatial Information Science **XXXIX-B4**, 281-286 (2012).

22 Batt, G. E., Braun, J., Kohn, B. P. & McDougall, I. Thermochronological analysis of the dynamics of the Southern Alps, New Zealand. *Geological Society of America Bulletin* **112**, 250-266 (2000).

23 Kamp, P. J. J., Green, P. F. & White, S. H. Fission track analysis reveals character of collisional tectonics in New Zealand. *Tectonics* **8**, 169-195 (1989).

24 Little, T. A., Cox, S., Vry, J. K. & Batt, G. Variations in exhumation level and uplift rate along the obliqu-slip Alpine fault, central Southern Alps, New Zealand. *Geological Society of America Bulletin* **117**, 707-723 (2005).

25 Wellman, H. W. An uplift map for the South Island of New Zealand, and a model for uplift of the Southern Alps. *The Royal Society of New Zealand Bulletin* **18**, 13-20 (1979).

26 Enkelmann, E., Ehlers, T. A., Zeitler, P. K. & Hallet, B. Denudation of the Namche Barwa antiform, eastern Himalaya. *Earth and Planetary Science Letters* **307**, 323-333 (2011).

27 Herman, F., Rhodes, E. J., Braun, J. & Heiniger, L. Uniform erosion rates and relief amplitude during glacial cycles in the Southern Alps of New Zealand, as revealed from OSL-thermochronology. *Earth and Planetary Science Letters* **297**, 183-189 (2010).

28 Hovius, N., Stark, C. P. & Allen, P. A. Sediment flux from a mountain belt derived by landslide mapping. *Geology* **25**, 231-234 (1997).

29 Willett, S. D., Fisher, D., Fuller, C., En-Chao, Y. & Chia-Yu, L. Erosion rates and orogenic-wedge kinematics in Taiwan inferred from fission-track thermochronometry. *Geology* **31**, 945-948 (2003).

30 Barrows, T. T., Lehman, S. J., Fifield, L. K. & De Deckker, P. Absence of Cooling in New Zealand and the Adjacent Ocean During the Younger Dryas Chronozone. *Science* **318**, 86-89 (2007).
